# Inequality in Childhood Immunization Coverage: A Scoping Review of Data Sources, Analyses, and Reporting Methods

**DOI:** 10.3390/vaccines12080850

**Published:** 2024-07-29

**Authors:** Carrie Lyons, Devaki Nambiar, Nicole E. Johns, Adrien Allorant, Nicole Bergen, Ahmad Reza Hosseinpoor

**Affiliations:** Department of Data and Analytics, World Health Organization, 20 Avenue Appia, 1211 Geneva, Switzerland; lyonsc@who.int (C.L.); nambiard@who.int (D.N.); johnsn@who.int (N.E.J.); alloranta@who.int (A.A.); bergenn@who.int (N.B.)

**Keywords:** health inequalities, infant and child health, immunization, vaccination, scoping review

## Abstract

Immunization through vaccines among children has contributed to improved childhood survival and health outcomes globally. However, vaccine coverage among children is unevenly distributed across settings and populations. The measurement of inequalities is essential for understanding gaps in vaccine coverage affecting certain sub-populations and monitoring progress towards achieving equity. Our study aimed to characterize the methods of reporting inequalities in childhood vaccine coverage, inclusive of the settings, data source types, analytical methods, and reporting modalities used to quantify and communicate inequality. We conducted a scoping review of publications in academic journals which included analyses of inequalities in vaccination among children. Literature searches were conducted in PubMed and Web of Science and included relevant articles published between 8 December 2013 and 7 December 2023. Overall, 242 publications were identified, including 204 assessing inequalities in a single country and 38 assessing inequalities across more than one country. We observed that analyses on inequalities in childhood vaccine coverage rely heavily on Demographic Health Survey (DHS) or Multiple Indicator Cluster Surveys (MICS) data (39.3%), and papers leveraging these data had increased in the last decade. Additionally, about half of the single-country studies were conducted in low- and middle-income countries. We found that few studies analyzed and reported inequalities using summary measures of health inequality and largely used the odds ratio resulting from logistic regression models for analyses. The most analyzed dimensions of inequality were economic status and maternal education, and the most common vaccine outcome indicator was full vaccination with the recommended vaccine schedule. However, the definition and construction of both dimensions of inequality and vaccine coverage measures varied across studies, and a variety of approaches were used to study inequalities in vaccine coverage across contexts. Overall, harmonizing methods for selecting and categorizing dimensions of inequalities as well as methods for analyzing and reporting inequalities can improve our ability to assess the magnitude and patterns of inequality in vaccine coverage and compare those inequalities across settings and time.

## 1. Introduction

Vaccine development and distribution for children has contributed to improved childhood survival and health outcomes globally [1,2,3,4]. Immunization through vaccines in childhood serves not only as a vital intervention for disease prevention for individuals but also as an effective community intervention for controlling infectious diseases among populations. Unfortunately, progress in childhood vaccination coverage in the last decade has stalled, with over 14 million children worldwide remaining completely unvaccinated and substantially higher levels of children not receiving all recommended vaccines [5,6,7,8,9]. In response, the Immunization Agenda 2030 (IA2030) was developed as a global strategy for vaccines and immunization coverage [10].

Vaccine coverage among children is unevenly distributed across settings and populations. Globally, 60% of those children who have not received any vaccines reside in 10 low- or middle-income countries (Nigeria, India, Pakistan, Indonesia, Ethiopia, the Philippines, the Democratic Republic of Congo, Brazil, Angola, and Vietnam). This is indicative of inequalities, or observable differences, in vaccine coverage between countries [8]. Variations in vaccine coverage have also been widely observed within countries. In low- and middle-income countries, inequalities in vaccination coverage have been associated with socioeconomic status, rural vs. urban residence, and maternal education [11]. Although high-income countries generally have higher overall vaccine coverage, inequalities in childhood vaccination based on socioeconomic status have also been observed [12,13]. Across settings, marginalized or devalued communities are consistently disproportionately unvaccinated and under-vaccinated [14]. Not only do inequalities experienced in early childhood result in adverse health outcomes for children, but these inequalities will also likely be perpetuated or amplified throughout the life course. Given the existing evidence on inequalities in vaccination coverage and the implications for health outcomes, IA2030 has incorporated goals for the reduction in global inequities in vaccine coverage [10]. Specifically, IA2023 aims to extend immunization services to under-immunized children and communities and improve immunization coverage nationally and sub-nationally in a sustainable manner.

The measurement of inequalities allows for the understanding of the magnitude, context, and trends in inequalities across settings and populations and is also essential for monitoring progress towards achieving equity [15,16,17]. However, the methods used to assess and report inequalities vary, given the diverse data available, the multiple disciplines conducting research on inequalities, and the wide range of potential audiences and applications for inequality evidence [18]. Social inequalities measure how a health indicator varies between subgroups, which are defined by different dimensions of inequality such as socio-economic status. Inequalities can be assessed quantitatively using disaggregated data or summary measures, which can capture either absolute or relative inequalities. The World Health Organization recommends reporting both absolute and relative measures for tracking health inequalities [19,20,21,22,23]. The literature about the use and application of health inequality summary measures highlights that the selection of measures may influence the interpretation of results about trends over time and the level of inequalities based on settings, populations, or health conditions [24,25,26]. A systematic review found that health inequalities overall are most commonly reported using only relative measures, although this has not been formally assessed specifically for childhood vaccine coverage inequalities [27]. The ability to improve childhood immunization relies on having an accurate and comprehensive understanding of inequalities affecting populations based on socioeconomic, demographic or geographic dimensions. Therefore, assessing the landscape of inequality analyses and reporting for childhood vaccination will provide insight into the quality of evidence and identify opportunities for improvement.

In response, the objectives of the paper are to characterize the methods of reporting inequalities in childhood vaccine coverage, inclusive of the settings, data source types, analysis methods, and reporting modalities used to quantify and communicate inequality.

## 2. Materials and Methods

### 2.1. Protocol Development

We conducted a scoping review of publications in academic journals which included analyses of inequalities in vaccine coverage among children. The search strategy was developed using Medical Subject Headings (MeSH) and key terms focused on three concepts related to inequality, immunization, and children/infants. The literature searches were conducted in PubMed and Web of Science. The protocol was developed in adherence with the JBI Manual for Evidence Synthesis, and we followed the Preferred Reporting Items for Systematic reviews and Meta-Analyses extension for Scoping Reviews (PRISMA-ScR) Checklist [28,29]. This protocol can be accessed in File S1.

### 2.2. Inclusion and Exclusion Criteria

Articles were included if published between 8 December 2013 and 7 December 2023. Studies were eligible for inclusion if the study population was children under 5 years of age; the outcome was vaccine coverage; inequalities in vaccine coverage were examined by one or more socioeconomic, demographic, or geographic dimension; and the study reported an objective of assessing inequalities. No language restrictions were applied; however, only English search terms were used within primarily English databases. The full inclusion and exclusion criteria are included in Table 1.

### 2.3. Screening Process

**Figure 1:**
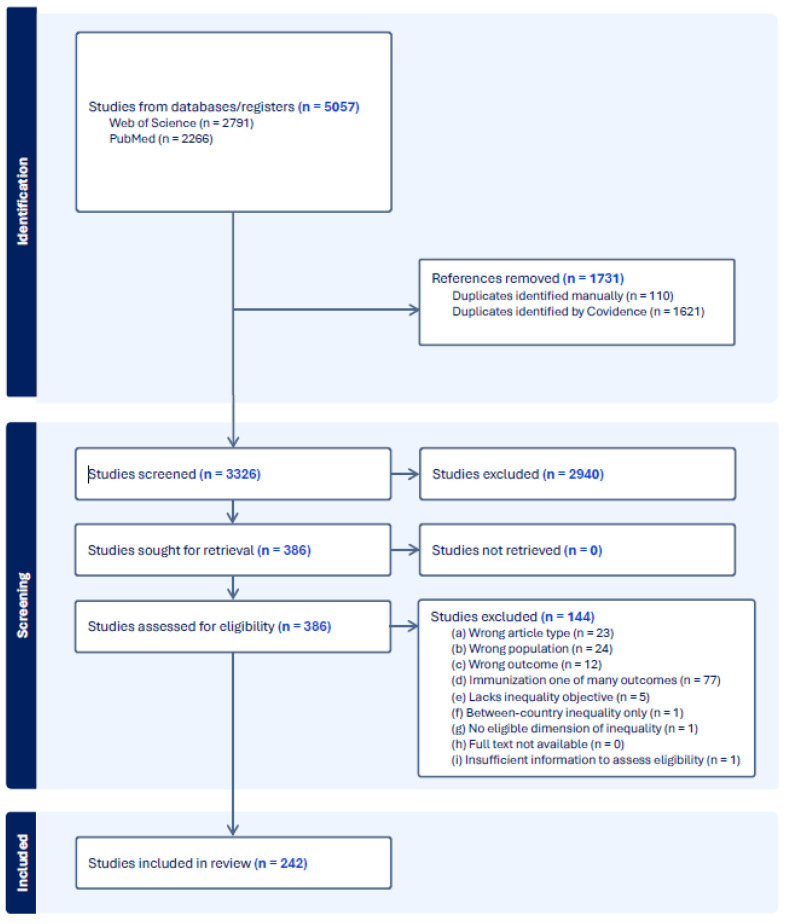
PRISMA flow diagram showing literature identification and screening.

The results from the literature search were reviewed using Covidence software (www.covidence.org, accessed on 18 March 2024) [30]. Title and abstract screening were conducted by one researcher (NJ), and the full text review was conducted by three reviewers (NJ, CL, AA). At the full text review stage, conflicts between reviewers were settled by a separate reviewer. Data extraction was carried out by four reviewers (NJ, CL, AA, DN). The flow chart of the review process is outlined in Figure 1. 

### 2.4. Data Extraction and Synthesis

The data extraction tool was developed using the Covidence data extraction template. The template was designed to extract basic study characteristics, outcomes, dimensions of inequality, and results.

The summary measures of inequality used in this review are defined based on the World Health Organization Health Equity Assessment Toolkit [26]. Dimensions of inequality were reported using PROGRESS-Plus-defined categories of place; race, ethnicity, culture, and language; occupation; gender and sex; religion; education; socioeconomic status; social capital; and additional context-specific dimensions, such as subnational region [31]. Data on the specific vaccines for each article were extracted, as well as outcomes reporting full vaccination (either of a single vaccine or multiple vaccines); vaccination initiation (at least one dose); non-vaccination (with one vaccine or multiple vaccines) or zero-dose (as defined by the study); drop-out, partial vaccination, or incomplete vaccination; and age-appropriate vaccination receipt. Data sources for vaccine outcomes were identified and extracted.

Demographic and Health Surveys (DHS) and/or Multiple Indicator Cluster Surveys (MICS) are large multi-country household surveys that provide publicly available data. The DHS Program is funded primarily through the United States Agency for International Development (USAID) and is designed to collect key population, health, and nutrition information for the entire population of a country. MICS are implemented through support by UNICEF and designed specifically to assess the health of women and children. Additional data sources were administrative surveys and other surveys including households or schools.

We described the number of publications over time and the distribution of studies using different data sources on vaccine coverage indicators over time. We further explored the landscape of publications selected in our review using Multiple Correspondence Analysis (MCA), which is a data visualization technique used to identify and illustrate underlying patterns in categorical data [32]. Each axis represents a dimension along which the data variability is maximized. Typically, the first two axes capture the most significant patterns of variation among the variables. MCA was performed using the FactoMineR package version 2.11 in R version 4.4.1.

## 3. Results

A total of 5057 potential studies were identified through the literature search, and 1731 duplicates were removed before screening. Titles and abstracts for 3326 studies were screened. Of these, 386 met the inclusion criteria for full-text evaluation. Finally, 242 studies underwent extraction. The full list of articles included is in Table S1.

**Figure 2:**
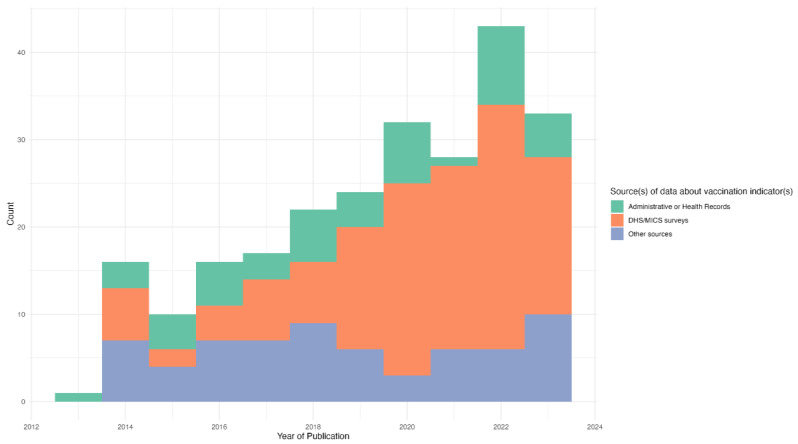
Publications of studies of inequalities in childhood vaccine coverage between 2013 and 2023 by data source for vaccine indicators (N = 242).

Overall, we observed an increase in the number of publications on inequalities in childhood vaccine coverage over the period of this review (Figure 2). There was a peak of 42 publications in 2022, noting that the literature search for years 2013 and 2023 did not include the entire calendar year. Differential increases in publications on inequalities in childhood vaccination by the data source of the vaccine indicators were observed over the review period. The number of publications using administrative or health surveillance data was relatively constant over the period, as were the publications based on other sources such as non-routine, study-specific, or small-scale surveys. However, publications utilizing DHS/MICS have increased in absolute numbers, as well as the overall proportion of manuscripts published since 2019. Across the entire period of the review, a total of 95 (39.3%) papers had utilized DHS/MICS data.

We described clusters of studies based on different categorical attributes in the MCA presented in Figure S1. Broadly, we observed a cluster of studies from low-income and lower-middle-income countries utilizing data from DHS/MICS. These studies primarily focus on full vaccination and zero-dose scenarios. In contrast, we observed another cluster of studies from high-income countries that use cohort data derived from administrative records.

Across all studies identified in this review (see Table 2), 15.7% (N = 38) of those included were multi-country studies, ranging from comparative studies of two countries within [33] or across regions [33,34] to a study that included 95 low- and middle-income countries [35] (Table 2). Most studies were cross-sectional in design (82.3% of all studies), followed by cohort (N = 39; 16.1%), as well as one study which was a randomized controlled trial. Of the studies with cohort study design, 87.2% of these used routine or administrative data sources (34 out of 39 studies). Most studies utilized DHS or MICS (53.3% of all studies) as well as survey data (25.6% of all studies) for the vaccine outcome. The study population was reported as the general population for 72.7% (N = 176) of all studies, while approximately one in five studies (22.3%) were focused on specific geographic regions. We also found that 223 out of 242 studies (92.1%) measured individual vaccine coverage as the outcome, while 15 studies (6.2%) computed vaccine coverage in small area units.

A range of indicators were used to characterize childhood vaccination. Over two-thirds of the studies (N = 163/242; 67.4%) used a single indicator, while the remaining used multiple indicators to report on vaccination. Overall, we found that the most commonly reported vaccine indicator was full coverage of multiple vaccines (58.3%), such as the coverage of WHO-recommended basic vaccine doses or the coverage of all countries’ Essential Programme on Immunization-recommended vaccine doses. The second most common indicator, reported in 36.8% of studies, was full vaccination of a specific vaccine series, including pentavalent or DTP vaccines (57 studies), measles or MMR vaccines (50 studies), and polio vaccines (42 studies). Drop-out was reported as an outcome in just under 15% of studies. Notably, zero-dose or non-vaccination was reported in about a fifth of studies, appearing more prominently in studies published after 2022 [36,37]. The most common type of vaccine (78.5%, 190/242) analyzed among studies was the pentavalent (Diphtheria, Pertussis, Tetanus, Hepatitis B, and Hib) vaccine or DPT (Diphtheria, Pertussis, and Tetanus) vaccine.

We did not see great variation in the summary measures used to characterize inequality (see Table 3). The most common analysis was a regression-based measure: odds ratios resulting from multivariate or multivariable logistic regression (N = 150; 62% of studies). A third of studies (36.8% N = 89) employed simple summary measures of health inequality: over a quarter (25.6%; N = 62) of studies reported the ratio, while over one in ten (11.2%; N = 27) reported the difference. Relative Concentration Index measures were reported in 19.8% of studies (N = 48). Overall, 19.0% (N = 46) studies reported absolute summary measures; 50.0% (N = 212) reported relative summary measures; and about 8.3% (N = 20) reported both absolute and relative summary measures. For studies that reported more than one summary measure, all were included in Table 2.

### 3.1. Single-Country Studies

**Figure 3:**
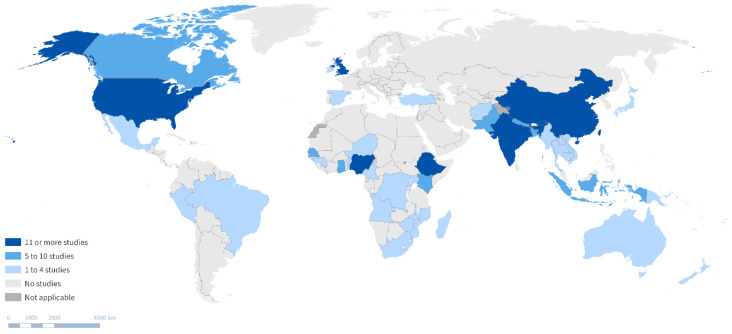
Global map of countries where studies on inequalities in childhood vaccination have been conducted between 2013 and 2023.

A total of 204 publications reported on inequalities in childhood vaccination in a single-country context (see Figure 3). Across these studies, 46 countries were represented. India had the largest number of papers, with a total of 34 publications in this period, followed by Ethiopia (N = 16), United Kingdom (N = 14), United States (N = 14), China (N = 12), and Nigeria (N = 12). Countries including Canada, Bangladesh, Ghana, and Nepal were featured in 5–10 publications in this period. We also found that 19 other countries had a single study published on this topic in the period studied.

In single-country studies, the most commonly reported dimension of inequality overall was socioeconomic status (71.9% of studies; see Table 4). The measures used to define socioeconomic status varied based on whether the variable was measured at the individual, household, regional, or country level. Of these studies, the most commonly reported measure for socioeconomic status was the wealth index, which was treated as a quantile, as a tertile, and as continuous. Other proxies for socioeconomic status were personal income, type of household, deprivation index, poverty level, and GDP per capita at the subnational level.

The next more commonly reported dimension of inequality overall was maternal education (67.5% of studies), followed by child’s sex (65.0%). This pattern was seen in countries across World Bank classification categories, although among high-income countries, there was a greater relative quantity of studies looking at race, ethnicity, culture, or language (58.7% of studies). Further, vulnerability indices were much more commonly applied in high-income country contexts. Religion as a dimension of inequality was much more commonly used in low- (N = 8) and lower-middle-income (N = 48) countries compared to in upper-middle-income (N = 1) and high-income (N = 3) countries. Lastly, we found that about a quarter of the single-country papers (N = 50) looked at multiple dimensions of inequality, and 28 used multiple disaggregation of inequality dimensions.

We also found an increasing use of vaccination indicators that may serve as proxies of inequity and disadvantage: zero-dose or non-vaccination was measured in 34 (or 16.7% of single-country) studies. Among these studies, the most commonly reported dimension of inequality was maternal education (76.5% of single-country studies reporting non-vaccination or zero-dose), followed closely by socioeconomic inequality (67.6% of single-country studies reporting non-vaccination or zero-dose), place of residence (64.7% of single-country studies reporting on this indicator), as well as gender and sex (58.5% of single-country studies reporting on this indicator). No studies examining zero-dose or non-vaccination utilized a vulnerability index. The findings broadly matched the patterns seen in full vaccine coverage studies, meaning that higher education and socio-economic statuses were associated with a lower zero-dose prevalence. Further, minoritized racial and ethnic groups, as well as religious groups, reported a higher zero-dose prevalence. However, for certain dimensions of inequality, like gender and sex as well as place of residence, a number of studies reported no association or associations that became insignificant in the multivariate analysis. The full results of the evidence for single-country studies are included in Table S2.

### 3.2. Multi-Country Studies

Of the 38 multi-country studies identified in our review, the most commonly reported dimension of inequality was socioeconomic status, defined as the wealth index, household wealth, or household disposable income. Of the multi-country studies, 20 had data on full coverage of multiple vaccines. Among multi-country studies, 94.7% (N = 36) used DHS/MICS data. Four studies (10.5%) used multiple disaggregation, and over a quarter of the studies (N = 10) looked at inequality trends over time. Finally, subnational inequalities in vaccination were reported in a number of multi-country studies [38,39,40]. The full results of evidence for single-country studies are included in Table S3.

## 4. Discussion

Our scoping review aimed to characterize the methods of reporting inequalities in childhood vaccine coverage, inclusive of the settings, data source types, analysis methods, and reporting modalities used to quantify and communicate inequality. We observed that analyses on inequalities in childhood vaccination rely heavily on DHS or MICS data and that papers leveraging these data had increased in the last decade. We found that few studies analyzed and reported inequalities using summary measures and instead largely used multivariate or multivariable logistic regression models for analyses. The most commonly analyzed dimensions of inequality were economic status and maternal education, and the most common vaccine outcome indicator was full vaccination of multiple vaccines. However, the definition and construction of both dimensions of inequality and outcome measures varied across studies.

Summary measures allow inequalities to be described by a single number and can be useful in describing, monitoring, and comparing inequality across settings and over time [26]. However, a low proportion of studies identified in this review used summary measures of inequalities. Overwhelmingly, the studies used multivariable or multivariate logistic regression models. While these models provide valuable insights by simultaneously examining the influence of multiple factors on the outcome of interest, they do not strictly qualify as summary measures of inequality. Estimates from logistic regression provide an estimate of the direct effect of a dimension of inequality, while summary measures of inequality provide a measure of the total effect of a dimension of inequality. By accounting for various potential confounders, these models offer a more nuanced understanding of the association between socioeconomic status and vaccine coverage. However, their reliance on specific sets of covariates, which are tailored to the author’s conceptual framework and the available data, complicates the comparability of effect estimates between studies. This variability in model specifications can lead to inconsistencies in findings and hinder efforts to synthesize evidence across diverse research endeavors. Additionally, the construction of models may come with limitations, especially when not accounting for the study design and sampling approaches. For example, several studies identified in our review leveraged DHS or MICS data and included the place of residence but not regions in the regression models. DHS and MICS use both the place of residence and regions as strata for survey sampling and therefore oversample certain regions as needed [41,42]. Thus, if vaccine coverage is greater in certain regions than others, then this omission of both place of residence and regions as potential confounders would likely introduce a bias into the estimate. Multivariate and multivariable logistic regression models serve an important purpose; however, there is a need for more studies reporting summary measures to inform the monitoring and tracking of inequalities across countries and over time.

Among the studies identified in this review, approximately one in five reported absolute summary measures, while half reported relative summary measures and about one-tenth reported both absolute and relative summary measures [19,20,21,22,23]. The selection of reporting absolute vs. relative summary measures may influence the interpretation, conclusions, and implications of the study results [24,25,26]. For example, one study used multiple inequality summary measures, which all led to fairly consistent findings for the presence of inequalities but inconsistent findings for trends of inequality over time [43]. Although eight percent of studies reported both absolute and relative summary measures, there is an opportunity for more studies to leverage both types of measures to provide more meaningful and informative results and reduce reporting bias. Simple measures of inequality, such as the difference and ratio, were used in a large proportion of analyses identified in this review and allow for comparisons of vaccination outcomes in two population subgroups. However, the selection of only two subgroups may not reflect the level of inequalities across diverse subgroups in the population. Conversely, complex measures allow for comparison with more than two population subgroups and were used in about one-third of the studies identified in this review. Most of the complex summary measures reported in this review leveraged disproportionality measures such as the relative concentration index, which is a measure of inequality that shows the gradient across population subgroups and indicates the extent to which inequality is concentrated among certain subgroups.

Impact summary measures are used to estimate the potential benefits of addressing inequalities in childhood vaccination coverage. Overall, only 2% of studies (N = 4) identified in this review used impact measures—specifically, the population attributable fraction—to show the potential improvement that could be achieved if all population subgroups had the same level of vaccine coverage as a reference subgroup. Several other studies were focused on assessing the potential for inequality reduction. For example, one study used an equity outcome in program evaluation [44] and another study aimed at evaluating changes in inequalities as a result of a policy [45]. Although measuring the state of inequalities and monitoring inequalities are essential, there is a need for more studies to look forward towards how to reduce inequalities and improve childhood vaccination coverage.

This review highlighted that the most commonly analyzed dimensions of inequality were economic status and maternal education. However, the distribution of the use of these varied based on country-level income, as a higher proportion of studies in low- and middle-income settings utilized these dimensions than in high-income settings. Conversely, race and ethnicity were explored in a high proportion of studies in high-income settings compared to those in lower- and middle-income settings. The selection of dimensions of inequalities may reflect the differences in the drivers and conceptual pathways in inequalities in high-income settings compared to those in lower- and middle-income settings. Importantly, the definitions and categorizations of the dimensions differed, with over ten different ways in which economic status was defined and the measure was constructed. Studies may have leveraged data-driven or conceptual approaches for categorizing dimensions of inequalities; however, using conceptual approaches for categorization may allow for uniform measures across different settings and datasets.

Across studies identified in this scoping review, vaccine outcome indicators varied in how they were constructed. For example, the most commonly reported vaccine indicator was full vaccination of multiple vaccines. However, not all of these studies utilized the same set of vaccines in their definition of full vaccination coverage. Almost half of the studies in this review used non-vaccination as a vaccine outcome, and this was defined as non-vaccination with one specific vaccine, non-vaccination of multiple vaccines, or as ‘zero dose’ (children who have not received any routine vaccinations). The choice and construction of vaccine outcomes may influence the inequality observed, the interpretation of the results, and the utility of the findings. For example, analyzing full coverage in vaccination may provide insight into inequalities in subgroups who are not achieving the recommended vaccination coverage and provide insight into how programs may fill gaps in reaching goals for full coverage. Conversely, assessing inequalities in non-vaccination or zero coverage provides insights into the sub-populations that may be most marginalized and not obtaining any vaccine coverage, highlighting subgroups with a severe need for interventions.

Our scoping review highlights the patterns in data sources used for assessing inequalities among children. Across studies, DHS or MICS were the most commonly used data source for vaccine indicators. Importantly, the utilization of DHS or MICS appears to have increased over the last decade and is contributing to a larger proportion of the literature on inequalities in childhood vaccinations. Notably, almost all the multi-country studies utilized DHS or MICS data, highlighting the reliance on these data for regional and global analyses of inequalities. Therefore, the quality of this body of literature is heavily tied to the quality of these data. Both DHS and MICS are household surveys and are designed to be nationally representative samples. Given that DHS and MICS use consistent indicators across countries, and the recruitment methods are standardized, cross-country comparisons are feasible. Both DHS and MICS have publicly available datasets and are therefore accessible to researchers who wish to analyze and report on disaggregated data. However, there are some limitations of using these data, such as the lack of control over the selection and measurement of the dimensions of inequalities available. Additionally, there is generally a large lag in the release of data and reports from when the data are collected. Lastly, DHS/MICS may have limited data on marginalized populations of interest for inequality studies.

Our scoping review highlights that there are challenges in comparing results of inequalities in childhood vaccination across settings and over time. These challenges arise from the differences in the data used for analyses, indicator definitions for vaccinations as well and dimensions of inequalities, and summary measures. Although the goal of our review was not to summarize the evidence of studies, we explored the evidence in Table S2 for the purpose of understanding the patterns and potential implications of the methods used. Given the differences in the data, indicators, methods, and summary measures used, we expect to observe inconsistencies in the results across settings. For example, studies assessing residence using rural vs. urban settings as the dimension of inequality varied in terms of inequalities favoring either urban or rural. One may conclude that these findings highlight that rural–urban inequalities are likely very context- and setting-dependent and that this variation likely depends on the funding and programmatic priorities and efforts towards vaccination in either setting. However, the variation in results may alternatively be a product of the methods used for assessment, including definitions of vaccine coverage or of urbanicity, rather than actual inequalities. Despite challenges and limitations, some consistencies in the results were observed across studies. For example, maternal education and economic status are widely used dimensions of inequality for assessing vaccination among children, with more than half of the studies identified in this review analyzing at least one of these dimensions. Across studies, the results were largely consistent with inequalities favoring higher education and a higher economic status. This may highlight the persistent and universal role that education and economic statuses may serve in inequalities in childhood vaccines, regardless of the country, setting, or methods used. However, harmonizing methods used in assessing inequalities will improve our ability to accurately compare across countries, across populations, and over time.

There are several limitations that should be considered for this scoping review. The results from this search strategy are subject to how the manuscripts were indexed into each database. Therefore, relevant manuscripts may not have been detected in the search. For our search strategy, only predominately English-language databases were searched, and therefore, relevant articles in non-English databases may not have been identified in the search. Our search was limited to articles published in academic journals, and therefore, literature such as project reports, normative agency reports, or other studies may not have been identified in our search strategy.

## 5. Conclusions

Measuring and monitoring inequalities in childhood immunization is essential to achieving health equity. Currently, the evidence on inequalities in childhood vaccination in academic journals relies on the use of various approaches including data, analytical methods, and measures of results, which makes comparisons across settings and time difficult. The harmonization of approaches may allow for improved monitoring through academic studies.

## Figures and Tables

**Table 1:** Inclusion and exclusion criteria for articles obtained through the search for inequality analyses in childhood vaccine coverage.

Inclusion Criteria	Exclusion Criteria
Study population of children under the age of 5 years. Peer-reviewed research articles and research reports with the use of primary or secondary data published in academic journals. Studies examining an outcome of vaccination coverage or lack of coverage, including dropout and partial/incomplete vaccination. Reporting vaccination coverage by one or more socioeconomic, demographic, or geographic dimension(s) of inequality. Assessed within-country inequality.	Study population did not include results specific to children <5 years. The following document types: Short communications, comments, letters, editorials, biographies, reference materials, interviews, conference proceedings, news articles, pre-prints and systematic, scoping, and other reviews. Studies that exclusively used a qualitative methodology. Articles published more than 10 years before the search date. Only evaluates inequalities in vaccination coverage by medical factors, diagnoses, or comorbidities. Study includes multiple childhood health or related development outcomes, of which immunization is only one (e.g., includes immunization, nutrition, and education outcomes). Evaluates only between-country inequalities. Full text is not available.

**Table 2:** Characteristics of studies on inequalities in childhood vaccination conducted between 2013 and 2023 (N = 242).

Country	N	%
Single-country	204	84.3%
Multi-country	38	15.7%
**Study design**		
Randomized control trial	1	0.4%
Cohort study	39	16.1%
Cross-sectional study	199	82.3%
Other	3	1%
**Sources of data for vaccine indicator**		
DHS or MICS	129	53.3%
Other surveys (household, school, etc.)	62	25.6%
Administrative	51	21.1%
Other	4	1.7%
**Vaccine indicator classification**		
Full vaccination of multiple vaccines	141	58.3%
Full vaccination of a specific vaccine	89	36.8%
Vaccination initiation (at least one dose of a multi-dose vaccine series)	27	11.2%
Non-vaccination (with one or multiple vaccines)/zero dose	45	19.0%
Drop-out, partial vaccination, or incomplete vaccination	36	14.9%
Age-appropriate vaccination receipt	16	6.6%
Other	8	3.3%

**Table 3:** Summary measures or effect estimates of inequality used in studies on inequalities in childhood vaccination conducted between 2013 and 2023.

Type of Summary Measures or Regession Method	N	%
Regression-based odds ratios	150	62.0%
Ratio	62	25.6%
Relative concentration index	48	19.8%
Difference	27	11.2%
Slope Index of Inequality	13	5.4%
Population Attributable Risk	6	2.5%
Relative Index of Inequality	4	1.7%
Population Attributable Fraction	5	2.1%
Index of Disparity	1	0.4%
Theil Index	1	0.4%

**Table 4:** Dimensions of inequality assessed and reported in single-country studies on inequalities in childhood vaccination conducted between 2013 and 2023 (N = 204).

	Overall (N = 204)		2023 World Bank Group Country Income Category							
			Low Income (N = 30)		Lower-Middle Income (N = 102)		Upper Middle Income (N = 26)		High Income (N = 46)	
PROGRESS-Plus characteristic	N	%	N	%	N	%	N	%	N	%
Place of residence (rural/urban)	118	58.1%	21	70.0%	71	69.6%	18	69.2%	8	17.4%
Race, ethnicity, culture, language	89	43.8%	6	20.0%	48	47.1%	8	30.8%	27	58.7%
Occupation (maternal)	53	25.6%	11	36.7%	30	29.4%	10	38.5%	2	4.3%
Gender and sex (Child’s sex)	133	65.0%	18	60.0%	79	77.4%	21	80.8%	15	32.6%
Religion	60	29.6%	8	26.7%	48	47.1%	1	3.8%	3	6.5%
Education (maternal)	138	67.5%	25	83.3%	84	82.42%	18	69.2%	11	23.9%
Socioeconomic status	146	71.9%	25	83.3%	76	75.2%	21	80.8%	24	52.2%
Subnational region	99	48.8%	16	53.3%	58	57.4%	10	38.5%	15	32.6%
+ Vulnerability index)	21	10.3%	0	0.0%	4	4.0%	1	3.8%	16	34.8%

## Data Availability

Not applicable.

## Supplementary Materials

The following supporting information can be downloaded at: https://www.mdpi.com/article/10.3390/vaccines12080850/s1, File S1: Search Protocol; Table S1. Full list of articles identified in the scoping review on inequality in childhood immunization coverage; Figure S1: Multiple Correspondence Analysis of Article Characteristics on Child Vaccine Coverage Inequalities; Table S2. Summary of findings of single-country studies on inequalities in child vaccine coverage conducted between 2013 and 2023; Table S3. Summary of findings of multi-country studies on inequalities in child vaccine coverage conducted between 2013 and 2023 [46,47,48,49,50,51,52,53,54,55,56,57,58,59,60,61,62,63,64,65,66,67,68,69,70,71,72,73,74,75,76,77,78,79,80,81,82,83,84,85,86,87,88,89,90,91,92,93,94,95,96,97,98,99,100,101,102,103,104,105,106,107,108,109,110,111,112,113,114,115,116,117,118,119,120,121,122,123,124,125,126,127,128,129,130,131,132,133,134,135,136,137,138,139,140,141,142,143,144,145,146,147,148,149,150,151,152,153,154,155,156,157].
