# Supplementary material for: Inequality in Childhood Immunization Coverage: A Scoping Review of Data Sources, Analyses, and Reporting Methods
Source: Vaccines (Basel). 2024 Jul 29;12(8):850. doi: 10.3390/vaccines12080850 (PMC11360733; doi:10.3390/vaccines12080850)
Supplement: Supplementary file 1 [file vaccines-12-00850-s001.zip › File S1.pdf]

Supplementary File S1: Review protocol, search strategy and data extraction template

1. **PROTOCOL**

Note: this protocol was developed in adherence with the JBI Manual for Evidence Synthesis (1).
Background: Despite significant progress in improving access to childhood immunizations, millions of children worldwide remain underimmunized or completely unvaccinated. These gaps in immunization coverage are inequitably distributed, concentrated in marginalized communities and among disadvantaged populations (2). The ways in which inequalities are measured and reported influence the ways in which readers and policymakers interpret and understand results. A review of the body of literature detailing the measurement, assessment, and reporting of immunization coverage equity is necessary to characterize the nature of methodologies used to assess and report inequality and identify opportunities for improvement in evaluations of inequality and communication of results.

Published reviews on similar topics:

- Several reviews summarize demographic and socioeconomic differences, or factors associated with inequalities in, childhood vaccination coverage (for example, Ali et al 2022, Bocquier et al 2017) (3, 4). These reviews summarize reported inequalities in coverage, largely finding that wealth, education, geographic access, and child gender are consistently associated with coverage. These reviews focus on quantifying the inequalities in coverage observed; we build upon these by summarizing the ways in which inequalities are evaluated and reported.
- A review by King et al (2012) summarized the use of relative and absolute measures in reporting health inequalities in a single peer-reviewed journal (5). They found that health inequalities were most commonly reported using only relative measures, and that greater understanding of the "magnitude, direction, significance, and implications" of inequalities could be gained from also reporting absolute measures. We expand upon this work by examining a broader range of publications and a narrower topic focus on childhood vaccines.
- Work by Schlotheuber and Hosseinpoor (2022) summarized available measures of inequality (6). This study illustrated the numerous measures that can be applied, and how different measures offer different insights into understanding inequalities. This study summarized measurement of inequality but did not review the literature for use of these measures.
- A scoping review by Bergen et al (2023) assessed the reporting of socioeconomic, demographic, and geographic inequalities in COVD-19 immunization (7). Findings suggested that a range of inequality dimensions were explored, most frequently age, race/ethnicity, and sex/gender, and that fully half of the included studies were conducted in the United States. The structure of this work serves as a model for our assessment of reporting of inequality in childhood vaccines specifically.

Central question: What is the current landscape of the published literature which aims to directly report on, assess, or evaluate inequalities in childhood immunization?

Review objectives: The objectives of the paper are to summarize the nature and methods of reporting inequalities in childhood immunization, inclusive of the settings, data types, analysis methods, and reporting modalities used to quantify and communicate inequality.

Scoping review methods

Justification
The proposed paper will be a scoping review. Scoping reviews are appropriate "to determine the scope or coverage of a body of literature on a given topic and give clear indication of the volume of literature and studies available as well as an overview (broad or detailed) of its focus. Scoping reviews are useful for examining emerging evidence when it is still unclear what other, more specific questions can be posed and valuably addressed by a more precise systematic review" (8).
Inclusion and exclusion criteria

1. **Included population**: The work must pertain to human populations under age 5.

2. **Excluded population:** Work exclusively examining animal models or children 5 and over, adolescents, or adults will be excluded. Studies which only present modeling/projection of human populations will also be excluded.
3. **Included article types.** Peer reviewed research articles and research reports reporting primary or secondary data will be considered for inclusion. Brief reports and short research articles (5 pages or less) will be considered for inclusion; if included, these types of articles are marked as "short research papers". Articles must have been published within 10 years of the search date.
4. **Excluded article types**. The following document types will be excluded: Short communications, comments, letters, editorials, biographies, reference materials, interviews, conference proceedings, news articles, pre-prints. Systematic, scoping, and other reviews will be excluded. Studies which exclusively use a qualitative methodology will be excluded. Articles published more than 10 years before the search date will be excluded.
5. **Included outcomes.** The work must pertain to childhood vaccinations received by children 5 or younger. Only documents examining the outcome of coverage or lack of coverage will be included. The outcomes of dropout and partial/incomplete vaccination will also be included as these are considered measures of lack of coverage.
6. **Excluded outcomes.** Documents will be excluded if they exclusively examine the following outcomes: vaccines received by older children, adolescents, or adults; intent to vaccinate, vaccine hesitancy, vaccine refusal, or reasons for non-vaccination; composite child and/or maternal health indicators; immunity among children not derived from direct vaccination (e.g. through maternal immunization or naturally acquired immunity) or of unknown source (e.g. seroprevalence as outcome); the nature of vaccine receipt or recall (including location or cost of vaccination services or method of coverage recall); missed opportunities for vaccination; time of vaccination receipt, including timely, age-appropriate, or delayed vaccination. Documents will be excluded if they examine immunization as only one indicator of services or health outcomes more broadly; e.g. if the primary objective of the manuscript is to examine RMNCH service utilization, healthcare utilization, preventive service uptake, or MDG / SDG indicators.
7. **Study objective.** Documents will be considered for inclusion if they contain an objective or focus pertaining to quantitatively reporting, measuring, summarizing, or evaluating inequality in child immunization coverage within one or more national or subnational populations. Studies which do not report or analyze inequalities in coverage will be excluded. Studies which only examine inequalities in national-level coverage between countries will be excluded.
8. **Dimensions of inequality.** Documents will be considered for inclusion if they report vaccination coverage by one or more socioeconomic, demographic or geographic dimensions of inequality. Documents will be excluded if they only evaluate inequalities in vaccination coverage by medical factors, diagnoses, or comorbidities.
9. **Language.** No language exclusion will be applied; however, searches will be performed using only English search terms in primary English databases (PubMed and WoS).
10. **Full text availability.** For inclusion, the full text of the document must be available.

Search strategy
Literature searches will be conducted systematically in PubMed and Web of Science. Searches will be limited to publications published in the past 10 years from date of search. No language or article type restrictions will be applied. The search strategy will consist of three domains related to 'equity' AND 'immunization' AND ('child' OR 'infant') search terms.

Study selection methods
The results from the literature search will be imported to Covidence software. Title and abstract scan will be conducted by one researcher, Nicole Johns (NJ). This will be followed by full text review by two reviewers, including two of NJ, Carrie Lyons (CL), Adrien Allorant (AA), in consultation with Ahmad Reza Hosseinpoor (ARH), as needed to reach agreement for inclusion/exclusion. For studies excluded during the full text review, the first reason for exclusion will be recorded, according to the following ordered list: (a) wrong article type; (b) does not pertain to children under 5/vaccination coverage occurring before age 5; (c) does not have an outcome of immunization coverage, lack of coverage, dropout, or incomplete or partial vaccination; (d) does not focus solely or explicitly on immunization as an outcome; (e) does not have a study objective or focus of reporting, measuring, summarizing, or evaluating inequality; (f) only reports on between-country inequality; (g)

does not meet criteria for dimension of inequality; (h) full text not available; (i) insufficient information to assess eligibility.

Data extraction and synthesis

A data extraction template will be applied to extract relevant information from each included article. This includes: general information about the article and where it was published; characteristics of the study setting, population, study objective and design; characteristics of the vaccination indicator; characteristics of the dimensions of inequality; analysis methods including whether disaggregation, summary measures of inequality, multivariate regression, or other approaches were utilized; and reporting of inequality results, including whether text, figures, tables, or interactive results were shared as part of either the main text or supplementary materials and whether and how uncertainty was reported.

To the extent possible, the information will be copied from the original source into the data extraction template, and then tabulated across studies, noting where any terminology or concepts may be combined or adapted (e.g. tabulating dimensions of inequality addressing 'economic status' which may encompass different measurement approaches). After reviewing studies in tandem to ensure consistency in how the template is interpreted and applied, one researcher will do the data extraction for each included study. The researchers will periodically consult and reach consensus on any questions or points of ambiguity that arise.

References

1. Peters MDJ, Godfrey C, McInerney P, Munn Z, Tricco AC, Khalil H. Chapter 11. Scoping reviews. In: Aromataris E, Munn Z, editors. JBI manual for evidence synthesis [Internet]. JBI; 2020 [cited 2023 Jan 3]. Available from: https://jbi-global-wiki.refined.site/space/MANUAL/4687342/Chapter+11%3A+Scoping+reviews
2. Immunization Agenda 2030: A global strategy to leave no one behind. https://www.immunizationagenda2030.org/images/documents/VisionStratagiy/BLS20116_IA_Visual-ID-DesignLayout_spread_009_WEB.pdf
3. Ali HA, Hartner AM, Echeverria-Londono S, Roth J, Li X, Abbas K, Portnoy A, Vynnycky E, Woodruff K, Ferguson NM, Toor J. Vaccine equity in low and middle income countries: a systematic review and meta-analysis. *International Journal for Equity in Health*. 2022 Dec;21(1):1-30.
4. Bocquier A, Ward J, Raude J, Peretti-Watel P, Verger P. Socioeconomic differences in childhood vaccination in developed countries: a systematic review of quantitative studies. *Expert review of vaccines*. 2017 Nov 2;16(11):1107-18.
5. King NB, Harper S, Young ME. Use of relative and absolute effect measures in reporting health inequalities: structured review. *BMJ*. 2012 Sep 3;345.
6. Schlotheuber, Anne, and Ahmad Reza Hosseinpoor. "Summary measures of health inequality: A review of existing measures and their application." *International Journal of Environmental Research and Public Health* 19.6 (2022):3697.
7. Bergen N, Johns NE, Chang Blanc D, Hosseinpoor AR. Within-Country Inequality in COVID-19 Vaccination Coverage: A Scoping Review of Academic Literature. *Vaccines*. 2023 Feb 23;11(3):517.
8. Munn Z, Peters MDJ, Stern C, Tufanaru C, McArthur A, Aromataris E. Systematic review or scoping review? Guidance for authors when choosing between a systematic or scoping review approach. *BMC Med Res Methodol*. 2018 Dec;18 (1):143.

Search terms

- Inequality
  - MeSH Terms: Health Equity, Healthcare Disparities, Health Status Disparities, Health Inequities
  - Title/abstract/topic terms: equit*, inequit*, inequal*, equal*, disparit*, deprivation, disadvantage*, underserved, concentration index, social class*, social position
- Immunization
  - MeSH Major Topic: Vaccination, Vaccination Coverage, Immunization
  - MeSH Terms: vaccination/statistics and numerical data, vaccination/trends, vaccination coverage/statistics and numerical data, vaccination coverage/trends, immunization/statistics and numerical data, immunization/trends, immunization programs/statistics and numerical data, immunization programs/trends
  - Title/abstract/topic terms: vaccin*, immuniz*, immunis*
- Child

- MeSH Terms: Infant; Infant, Newborn; Child; Child, Preschool
- Title/abstract/topic terms: child*, infant, under-5, under 5, under-five, under five

- Limited to **past 10 years**

PubMed

(

("Health Equity"[MeSH Terms] OR "Healthcare Disparities"[MeSH Terms] OR "Health Status Disparities"[MeSH Terms] OR "Health Inequities"[MeSH Terms])

OR ("equit*"[Title/Abstract] OR "inequit*"[Title/Abstract] OR "inequal*"[Title/Abstract] OR "equal*"[Title/Abstract] OR "disparit*"[Title/Abstract] OR "deprivation"[Title/Abstract] OR "disadvantage*"[Title/Abstract] OR "underserved"[Title/Abstract] OR "concentration index"[Title/Abstract] OR "social class*"[Title/Abstract] OR "social position"[Title/Abstract])

)

AND (

("Vaccination Coverage"[MeSH Major Topic] OR "Vaccination"[MeSH Major Topic] OR “Immunization”[MeSH Major Topic] OR "vaccination/statistics and numerical data"[MeSH Terms] OR “vaccination/trends”[MeSH Terms] OR "vaccination coverage/statistics and numerical data"[MeSH Terms] OR "vaccination coverage/trends"[MeSH Terms] OR “Immunization/statistics and numerical data”[MeSH Terms] OR “Immunization/trends”[MeSH Terms] OR “Immunization Programs/statistics and numerical data”[MeSH Terms] OR “Immunization Programs/trends”[MeSH Terms])

OR ("vaccin*"[Title/Abstract] OR "immuniz*"[Title/Abstract] OR "immunis*"[Title/Abstract])

)

AND (

(“Infant”[MeSH Terms] OR “Infant, Newborn”[MeSH Terms] OR “Child” [MeSH Terms] OR “Child, Preschool” [MeSH Terms])

OR (“child*"[Title/Abstract] OR “infant "[Title/Abstract] OR “under-5"[Title/Abstract] OR “under 5"[Title/Abstract] OR “under-five"[Title/Abstract] OR “under five "[Title/Abstract])

)

AND (("2013/12/8"[Date - Publication] : "2023/12/7"[Date - Publication]))

Web of Science

((TS=equit*) OR (TS=inequit*) OR (TS=inequal*) OR (TS=equal*) OR (TS=disparit*) OR (TS=deprivation) OR (TS=disadvantage*) OR (TS=underserved) OR (TS=concentration index) OR (TS=social class*) OR (TS=social position))
AND ((TS=vaccin*) OR (TS=immuniz*) OR (TS=immunis*))
AND ((TS=child*) OR (TS=infant) OR (TS=under-5) OR (TS=under 5) OR (TS=under-five) OR (TS=under five))
With date range selected manually, 2013/12/8-2023/12/7

DATA EXTRACTION TEMPLATE

General information

Study ID

First author last name + publication year
(use "-1" etc if needed to avoid duplicate)
e.g. Tan2022, Tan2022-1
[text entry]

Lead author

First author last name
[text entry]

Year published (select)

- o 2013

… [list countries 2013-2023]

- o 2023
- o Not applicable or not known

[single choice]

Article title

[text entry]

Journal name

[text entry]

Article type (as assigned by journal)

optional
e.g. original article
[text entry]

Article type - generic classification (select)

Some discretion is required to distinguish between a full vs short research paper - consider the length (~5 pages or less could be considered short) and depth of information/discussion

- o Review
- o Full research paper
- o Short research paper
- o Other

[single choice]

General characteristics of study

Country in which study was conducted

(If multiple, list all separated by comma)
[text entry]

Setting where study was conducted

If national, state 'national'. Else, specify specific geographic setting
e.g. Missouri, London, Eastern Mediterranean Region
[text entry]

Year(s) in which data was collected

[text entry]

Child population type (select)

General population
Specific geographic region(s)
Daycare(s) or school(s)
Health facility(ies)
Other: __________

[checkbox]

Study population (specify)

Include relevant details about the population e.g. children presenting a XX hospital, students at XX preschool, residents in XX neighbourhood
[text entry]

Aim or objective, as stated in the paper (specify)

Where possible copy text from study (use quotations)
[text entry]

Study design (select)

- o Randomised controlled trial
- o Non-randomised experimental study
- o Cohort study
- o Cross sectional study
- o Case control study
- o Prevalence study
- o Case series
- o Case report
- o Economic evaluation
- o Other: ______________

[single choice]
Characteristics of vaccine indicator(s)
Description of indicator (quote from study)
Where possible copy text from study (use quotations)
[text entry]
Vaccine biological classification (select)
Specify which vaccine(s) were assessed

Pentavalent (DTP+HiB+Hep B) or DTP (including DTaP)
HiB [Haemophilus influenzae type b]
Hepatitis B
Polio (including OPV & IPV)
Measles
MMR [measles, mumps, rubella]
BCG [tuberculosis]
Pneumococcal [PCV]
Rotavirus
COVID-19
Yellow fever
Varicella
Malaria
Influenza
Respiratory Syncytial Virus (RSV)
Japanese Encephalitis
Other

[checkbox]
Vaccine indicator classification (select)
Specify criteria for how indicator is defined in the study

Full vaccination of multiple vaccines (as defined by study; e.g. 'all basic doses', or 'all EPI vaccines')
Full vaccination of specific vaccine (as defined by study)
Vaccination initiation (at least one dose)
'Zero-dose' (as defined by study)
Non-vaccination (with one or multiple vaccines)
Drop-out, partial vaccination, or incomplete vaccination
Age-appropriate vaccination receipt
Not stated or unclear
Other

[checkbox]
Source(s) of data about vaccination indicator (select)

DHS or MICS survey
Other survey
Administrative, health, or surveillance records

Not stated
Other: _____________
[checkbox]
Note about data source
[text entry]
Level of measurement: vaccination indicator (select)
state the level at which vaccination coverage is measured (small area e.g. aggregated to county or district level)
- o Individual
- o Household
- o Small area (specify below)
- o Other: ______________

[single choice]
Note about level of measurement: vaccination indicator
[text entry]
Characteristics of dimensions of inequality
Dimension(s) of inequality included in study (select)
Maternal age
Paternal age
Child age
Economic status (including income and wealth)
Maternal education level
Paternal education level
Family size/characteristics
Maternal marital status
Maternal occupation/job
Paternal occupation/job
Place of residence urban/rural
Child or family immigration status/country of origin
Race, ethnicity, culture, or language
Religion
Child sex or gender
Subnational region or area
Vulnerability index
Other: _____________
[checkbox]
Specifications about dimensions of inequality
Note criteria (listing subgroups generally or specifically) and any other relevant details. E.g ‘Age in months’ or ‘Maternal age 15-19 vs 20-54'.
Maternal age (specify)
[text entry]
Paternal age (specify)
[text entry]
Child age (specify)
[text entry]
Economic status (specify)
[text entry]
Maternal education level (specify)
[text entry]
Paternal education level (specify)
[text entry]
Family size/characteristics (specify)
[text entry]
Maternal marital status (specify)
[text entry]

Maternal occupation/job (specify)
[text entry]
Paternal occupation/job (specify)
[text entry]
Place of residence urban/rural (specify)
[text entry]
Child or family immigration status/country of origin (specify)
[text entry]
Race/ethnicity/culture/language (specify)
[text entry]
Religion (specify)
[text entry]
Child sex or gender (specify)
[text entry]
Subnational region or area (specify)
[text entry]
Vulnerability index (specify)
[text entry]
Other dimension of inequality (specify)
[text entry]
Multiple disaggregation? (select)

- o No
- o Yes (specify below)

[single choice]
Describe multiple disaggregation (specify)
[text entry]
Source(s) of data about dimensions of inequality (select)

Surveys
Administrative or surveillance records
Census
Not stated
Other: ______________

[checkbox]
Note about data source
[text entry]
Level of measurement: dimension of inequality (select)

- o Same as immunization indicator (leave other fields blank)
- o Individual
- o Household
- o Small area (specify below)
- o Other: _______________

[single choice]
Note about level of measurement: dimension of inequality
[text entry]
Inequality analyses
How are inequality data analyzed? (select)

Coverage/prevalence estimates by level of inequality dimension
Bivariate tests of association (chi-squared, correlation)
Summary measure(s) of inequality
Multivariate regression
Other: ______________

[checkbox]
[If summary measure of inequality] Which summary measures were used? (select)

Difference
Ratio
SII (slope index of inequality)

RII (relative index of inequality)
ACI (Absolute concentration index)
CI or RCI (Relative concentration index) - note that if it is only called ‘Concentration index’, it is likely RCI
BGV (Between group variance)
BGSD (Between group standard deviation)
COV (Coefficient of variation)
MDM (Mean difference from mean)
MDB (Mean difference from best group)
IDIS (Index of disparity)
TI (Theil index)
Gini coefficient
MLD (Mean log deviation)
PAR (Population attributable risk)
PAF (Population attributable fraction)
Other: _____________

[checkbox]

Are changes in inequality over time analyzed/presented? (select)

- Yes
- No

[single choice]

Notes about data analysis/presentation (including any particularly interesting or novel analyses or visualizations)

[text entry]

Results

Main conclusions (overall) (specify)

Where possible, copy text from abstract or results/discussion/conclusion sections (use quotations)

[text entry]

Dimension of inequality specific results

Maternal age

[text entry]

Paternal age

[text entry]

Child age

[text entry]

Economic status

[text entry]

Maternal education level

[text entry]

Paternal education level

[text entry]

Family size/characteristics

[text entry]

Maternal marital status

[text entry]

Maternal occupation/job

[text entry]

Paternal occupation/job

[text entry]

Place of residence urban/rural

[text entry]

Child or family immigration status/country of origin

[text entry]

Race, ethnicity, culture, or language

[text entry]

Religion

[text entry]
Child sex or gender
[text entry]
Subnational region or area
[text entry]
Vulnerability index
[text entry]
Other dimension of inequality
[text entry]
Conclusions
Identified gaps in research (specify)
Note any gaps in research that are mentioned
[text entry]
Implications of research [text entry]
