# Supplementary material for: Inequality in Childhood Immunization Coverage: A Scoping Review of Data Sources, Analyses, and Reporting Methods
Source: Vaccines (Basel). 2024 Jul 29;12(8):850. doi: 10.3390/vaccines12080850 (PMC11360733; doi:10.3390/vaccines12080850)
Supplement: Supplementary file 1 [file vaccines-12-00850-s001.zip › vaccines-3083542 - Supplementary Figure S1.pdf]

Figure S1: Multiple Correspondence Analysis of Article Characteristics on Child Vaccine Coverage Inequalities. methodologies and focus areas not easily categorized into the previous two groups**.**

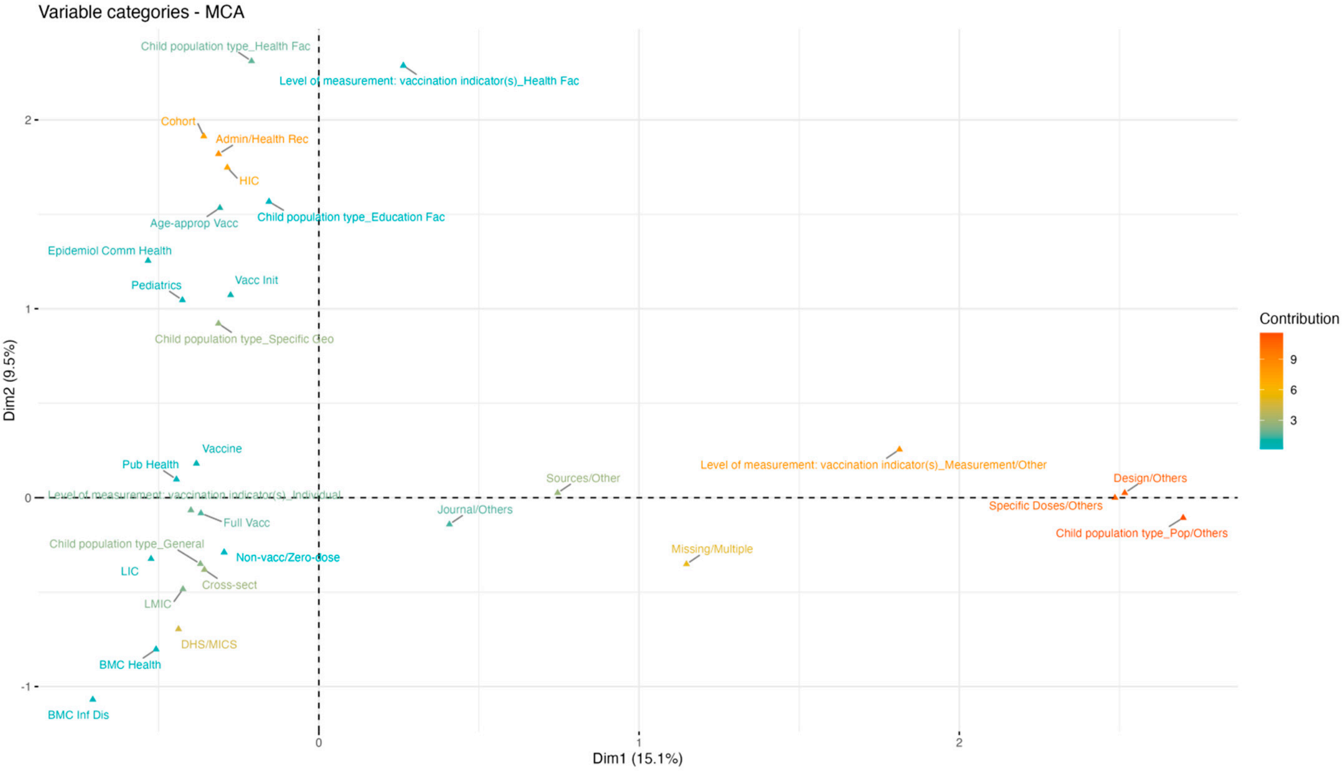

The plot illustrates the extent to which each variable contributes to the construction of these two axes. This contribution is quantified by how much of the inertia (a measure of variance in MCA) each variable accounts for in the axes. Figure S1 allows us to discern clusters of studies based on different categorical attributes. In the bottom left quadrant, we observe a cluster of studies from low-income and lower-middle-income countries (LIC/LMIC) utilizing data from Demographic and Health Surveys (DHS) and Multiple Indicator Cluster Surveys (MICS). These studies primarily focus on full vaccination and zero-dose scenarios. Contrastingly, the top left quadrant features studies from high-income countries that use cohort data derived from administrative records. Additionally, there appears to be a third group of more idiosyncratic studies scattered across other areas of the plot, indicating diverse methodologies and focus areas not easily categorized into the previous two groups. The variables used in the MCA included the journal name; the target population of the study (e.g., general population, adults, specific geography, daycare/school, etc.) ; the study design (e.g., cross-sectional, cohort study, etc.); the vaccine outcome indicator (e.g., full immunization, vaccination with a specific vaccine, etc.); the source of vaccination data and the source of inequality measurement data (e.g., DHS/MICS, administrative or health records); and the income group of the country or countries involved in the study. These variables were extracted during the scoping review process. MCA was performed using the FactoMineR package version 2.11 in R version 4.4.1.
