# Supplementary material for: Inequality in Childhood Immunization Coverage: A Scoping Review of Data Sources, Analyses, and Reporting Methods
Source: Vaccines (Basel). 2024 Jul 29;12(8):850. doi: 10.3390/vaccines12080850 (PMC11360733; doi:10.3390/vaccines12080850)
Supplement: Supplementary file 1 [file vaccines-12-00850-s001.zip › vaccines-3083542 - Supplementary Table S1.pdf]

Table S1. Full list of articles identified in scoping review on inequality in childhood immunization coverage

First author, year	Title
Acharya 2019	The trend of full vaccination coverage in infants and inequalities by wealth quintile and maternal education: analysis from four recent demographic and health surveys in Nepal.
Acharya 2022	Inequalities in full vaccination coverage based on maternal education and wealth quintiles among children aged 12-23 months: further analysis of national cross-sectional surveys of six South Asian countries.
Adebowale 2019	Relationship between household wealth and childhood immunization in core-North Nigeria.
Adedokun 2017	Incomplete childhood immunization in Nigeria: a multilevel analysis of individual and contextual factors.
Adokiya 2017	Evaluation of immunization coverage and its associated factors among children 12-23 months of age in Techiman Municipality, Ghana, 2016.
Aheto 2022	Multilevel analysis of predictors of multiple indicators of childhood vaccination in Nigeria.
Ahuja 2014	Gender inequalities in immunization of children in a rural population of Barabanki, Uttar Pradesh
Alaba 2021	Leaving No Child Behind: Decomposing Socioeconomic Inequalities in Child Health for India and South Africa.
Ali 2022	Factors associated with incomplete child immunization in Pakistan: findings from Demographic and Health Survey 2017-18.
Al-Kassab-Córdova 2022	Spatial distribution, determinants and trends of full vaccination coverage in children aged 12-59 months in Peru: A subanalysis of the Peruvian Demographic and Health Survey.
Al-Kassab-Córdova 2023	Inequalities in infant vaccination coverage during the COVID-19 pandemic: A population-based study in Peru.
Allan 2021	Inequities in childhood immunisation coverage associated with socioeconomic, geographic, maternal, child, and place of birth characteristics in Kenya.
Ameyaw 2021	Decomposing the rural-urban gap in factors associated with childhood immunisation in sub-Saharan Africa: evidence from surveys in 23 countries.
Anandappa 2018	Racial disparities in vaccination for seasonal influenza in early childhood.
Anderson 2022	Coverage, inequity and predictors of hepatitis B birth vaccination in Myanmar from 2011-2016: results from a national survey.
Arat 2020	Organisation of preventive child health services: Key to socio-economic equity in vaccine uptake?
Arat 2021	Childhood vaccination coverage in Australia: an equity perspective.
Arsenault 2017	Monitoring equity in vaccination coverage: A systematic analysis of demographic and health surveys from 45 Gavi-supported countries.
Ashbaugh 2018	Predictors of measles vaccination coverage among children 6-59â€¯months of age in the Democratic Republic of the Congo.
Asif 2019	Role of Maternal Education and Vaccination Coverage: Evidence From Pakistan Demographic and Health Survey.
Asif 2022	Decomposing socio-economic inequality in vaccination coverage among Pakistani children: A population-based cross-sectional study.
Asmare 2022	Disparities in full immunization coverage among urban and rural children aged 12-23 months in southwest Ethiopia: A comparative cross-sectional study.
Asresie 2023	Urban-rural disparities in immunization coverage among children aged 12-23 months in Ethiopia: multivariate decomposition analysis.
Asuman 2018	Inequalities in child immunization coverage in Ghana: evidence from a decomposition analysis.
Ataguba 2016	Explaining socio-economic inequalities in immunization coverage in Nigeria.
Atalell 2022	Mapping BCG vaccination coverage in Ethiopia between 2000 and 2019.

Atalell 2022	Spatial distribution of rotavirus immunization coverage in Ethiopia: a geospatial analysis using the Bayesian approach
Atteraya 2023	Inequalities in Childhood Immunisation in South Asia.
Bandara 2022	An equity-based assessment of immunization-related responses in urban Alberta during the 2014 measles outbreak: a comparative analysis between Calgary and Edmonton.
Bergen 2022	Economic-Related Inequalities in Zero-Dose Children: A Study of Non-Receipt of Diphtheria-Tetanus-Pertussis Immunization Using Household Health Survey Data from 89 Low- and Middle-Income Countries.
Bettampadi 2021	Vaccination Inequality in India, 2002-2013.
Bettampadi 2021	Impact of Multiple Risk Factors on Vaccination Inequities: Analysis in Indian Infants Over 2 Decades.
Bobo 2020	Decomposition of socioeconomic inequalities in child vaccination in Ethiopia: results from the 2011 and 2016 demographic and health surveys.
Bobo 2022	Child vaccination in sub-Saharan Africa: Increasing coverage addresses inequalities.
Boulton 2018	Socioeconomic factors associated with full childhood vaccination in Bangladesh, 2014.
Branco 2014	Socioeconomic inequalities are still a barrier to full child vaccine coverage in the Brazilian Amazon: a cross-sectional study in Assis Brasil, Acre, Brazil.
Brennan 2022	Does social deprivation correlate with meningococcal MenACWY, Hib/MenC and 4CMenB/Meningococcal Group B vaccine uptake in Northern Ireland?
Bryden 2019	The privilege paradox: Geographic areas with highest socio-economic advantage have the lowest rates of vaccination.
Budu 2022	Inequalities in the prevalence of full immunization coverage among one-year-olds in Ghana, 1993-2014.
Budu 2023	Socioeconomic and residence-based related inequality in childhood vaccination in Sub-Saharan Africa: Evidence from Benin.
Byrne 2018	Predictors of coverage of the national maternal pertussis and infant rotavirus vaccination programmes in England.
Cao 2018	Factors influencing the routine immunization status of children aged 2-3 years in China.
Carpiano 2019	Socioeconomic status differences in parental immunization attitudes and child immunization in Canada: Findings from the 2013 Childhood National Immunization Coverage Survey (CNICS).
Cata-Preta 2021	Patterns in Wealth-related Inequalities in 86 Low- and Middle-Income Countries: Global Evidence on the Emergence of Vaccine Hesitancy.
Cata-Preta 2022	Ethnic disparities in immunisation: analyses of zero-dose prevalence in 64 countries.
Charania 2018	Exploring immunisation inequities among migrant and refugee children in New Zealand.
Chartier 2018	Families First Home Visiting programme reduces population-level child health and social inequities.
Chidiebere 2014	Maternal sociodemographic factors that influence full child immunisation uptake in Nigeria
Chu 2022	Childhood immunization and age-appropriate vaccinations in Indonesia.
Clouston 2014	Social inequalities in vaccination uptake among children aged 0-59 months living in Madagascar: an analysis of Demographic and Health Survey data from 2008 to 2009.
Colomé-Hidalgo 2020	Monitoring inequality changes in full immunization coverage in infants in Latin America and the Caribbean.
Cortaredona 2020	Regional variations of childhood immunisations in Senegal: a multilevel analysis.
Cui 2013	Preventing hepatitis B though universal vaccination: reduction of inequalities through the GAVI China project.

Debie 2020	Complete vaccination service utilization inequalities among children aged 12-23â€‰months in Ethiopia: a multivariate decomposition analyses.
Debnath 2018	WEALTH-BASED INEQUALITY IN CHILD IMMUNIZATION IN INDIA: A DECOMPOSITION APPROACH.
Devasenapathy 2016	Determinants of childhood immunisation coverage in urban poor settlements of Delhi, India: a cross-sectional study.
Devkota 2016	Caste-ethnic disparity in vaccine use among 0- to 5-year-old children in Nepal: a decomposition analysis.
Dheresa 2021	Child Vaccination Coverage, Trends and Predictors in Eastern Ethiopia: Implication for Sustainable Development Goals
Dimitrova 2023	Essential childhood immunization in 43 low- and middle-income countries: Analysis of spatial trends and socioeconomic inequalities in vaccine coverage.
Dixon 2017	Vaccine uptake in the Irish Travelling community: an audit of general practice records.
Doherty 2014	Decomposing socioeconomic inequality in child vaccination: results from Ireland.
Donfouet 2019	Trends of inequalities in childhood immunization coverage among children aged 12-23 months in Kenya, Ghana, and CÃ´te d'Ivoire.
Donnelly 2022	Factors Influencing Health Equity of Influenza Vaccination in Pediatric Patients
Egondi 2015	Determinants of immunization inequality among urban poor children: evidence from Nairobi's informal settlements.
Eryurt 2022	Zero-dose children in Turkey: regional comparison of pooled data for the period 1990 to 2018.
Farrenkopf 2023	Understanding household-level risk factors for zero dose immunization in 82 low- and middle-income countries.
Fenta 2021	Determinants of full childhood immunization among children aged 12-23 months in sub-Saharan Africa: a multilevel analysis using Demographic and Health Survey Data.
Francis 2018	Factors associated with routine childhood vaccine uptake and reasons for non-vaccination in India: 1998-2008.
Fullman 2023	Assessing Potential Exemplars in Reducing Zero-Dose Children: A Novel Approach for Identifying Positive Outliers in Decreasing National Levels and Geographic Inequalities in Unvaccinated Children.
Gao 2020	Inequality in measles vaccination coverage in the "big six" countries of the WHO South-East Asia region.
Gebremedhin 2023	Oral and Inactivated Polio Vaccine Coverage and Determinants of Coverage Inequality Among the Most At-Risk Populations in Ethiopia.
Geweniger 2020	Childhood vaccination coverage and equity impact in Ethiopia by socioeconomic, geographic, maternal, and child characteristics.
Gilbert 2017	Determinants of non-vaccination and incomplete vaccination in Canadian toddlers.
Godin 2023	Municipality-level measles, mumps, and rubella (MMR) vaccine coverage and deprivation in Brazil: A nationwide ecological study, 2006 to 2020.
Goli 2020	Perplexing condition of child full immunisation in economically better off Gujarat in India: An assessment of associated factors.
Goodman 2023	Vaccination inequities among children 12-23Â months in India: An analysis of inter-state differences.
Gram 2014	Socio-economic determinants and inequities in coverage and timeliness of early childhood immunisation in rural Ghana.
Green 2015	Phased introduction of a universal childhood influenza vaccination programme in England: population-level factors predicting variation in national uptake during the first year, 2013/14.
Gutierrez 2023	Socioeconomic and geographic inequities in vaccination among children 12 to 59 months in Mexico, 2012 to 2021.
Hagedoorn 2023	Gaps in measles immunisation coverage for pre-school children in Aotearoa New Zealand: a cross-sectional study.

Haider 2019	Identifying inequalities in childhood immunisation uptake and timeliness in southeast Scotland, 2008-2018: A retrospective cohort study.
Hajizadeh 2018	Socioeconomic inequalities in child vaccination in low/middle-income countries: what accounts for the differences?
Hajizadeh 2019	Decomposing socioeconomic inequality in child vaccination in the Gambia, the Kyrgyz Republic and Namibia.
Hanifi 2018	Where girls are less likely to be fully vaccinated than boys: Evidence from a rural area in Bangladesh.
Harapan 2021	Religion and Measles Vaccination in Indonesia, 1991-2017.
Hardelid 2016	Factors associated with influenza vaccine uptake during a universal vaccination programme of preschool children in England and Wales: a cohort study.
Helleringer 2014	Polio supplementary immunization activities and equity in access to vaccination: evidence from the demographic and health surveys.
Herliana 2017	Determinants of immunisation coverage of children aged 12-59 months in Indonesia: a cross-sectional study.
Holipah 2020	Trends, Spatial Disparities, and Social Determinants of DTP3 Immunization Status in Indonesia 2004-2016.
Holroyd 2020	Characterizing mothers and children at risk of being under-immunized in India: A latent class analysis approach.
Hossain 2021	Trends and determinants of vaccination among children aged 06-59â€‰months in Bangladesh: country representative survey from 1993 to 2014.
Hosseinpoor 2016	State of inequality in diphtheria-tetanus-pertussis immunisation coverage in low-income and middle-income countries: a multicountry study of household health surveys.
Hu 2017	Determinants of inequality in the up-to-date fully immunization coverage among children aged 24-35 months: Evidence from Zhejiang province, East China.
Hu 2018	Inequities in Childhood Vaccination Coverage in Zhejiang, Province: Evidence from a Decomposition Analysis on Two-Round Surveys.
Hu 2018	Hepatitis B Vaccination among 1999â®»2017 Birth Cohorts in Zhejiang Province: The Determinants Associated with Infant Coverage.
Hu 2018	Analysis of the effects of individual- and community- level predictors on migrant children's primary immunization in Yiwu city, east China.
Hu 2019	Analyzing the Urban-Rural Vaccination Coverage Disparity through a Fair Decomposition in Zhejiang Province, China.
Hu 2021	The trends of socioeconomic inequities in full vaccination coverage among children aged 12-23 months from 2000 to 2017: evidence for mitigating disparities in vaccination service in Zhejiang province.
Hungerford 2016	Effect of socioeconomic deprivation on uptake of measles, mumps and rubella vaccination in Liverpool, UK over 16 years: a longitudinal ecological study.
Ijalba Martínez 2023	Acceptance and socioeconomic inequalities in meningococcal B vaccination in the community of Madrid prior to its inclusion in the immunization schedule.
Ikilezi 2020	Determinants of geographical inequalities for DTP3 vaccine coverage in sub-Saharan Africa.
Ilesanmi 2022	Trends, barriers and enablers to measles immunisation coverage in Saskatchewan, Canada: A mixed methods study.
Ishida 2022	Travel time to health facilities in Papua New Guinea: Implications for coverage and equity in child vaccinations.
Ishoso 2023	"Zero Dose" Children in the Democratic Republic of the Congo: How Many and Who Are They?
Jahan 2020	Changing trends in measles vaccination status between 2004 and 2014 among children aged 12-23 months in Bangladesh
Jammeh 2023	Comparing full immunisation status of children (0-23 months) between slums of Kampala City and the rural setting of Iganga District in Uganda: a cross-sectional study.

Joe 2015	Intersectional inequalities in immunization in India, 1992-93 to 2005-06: a progress assessment.
Johns 2022	Subnational Gender Inequality and Childhood Immunization: An Ecological Analysis of the Subnational Gender Development Index and DTP Coverage Outcomes across 57 Countries.
Johns 2022	Gender-Related Inequality in Childhood Immunization Coverage: A Cross-Sectional Analysis of DTP3 Coverage and Zero-Dose DTP Prevalence in 52 Countries Using the SWPER Global Index.
Johri 2021	Progress in reaching unvaccinated (zero-dose) children in India, 1992-2016: a multilevel, geospatial analysis of repeated cross-sectional surveys.
Joseph 2020	Spatial access inequities and childhood immunisation uptake in Kenya.
Joseph 2022	Understanding inequalities in child immunization in India: a decomposition approach
Juon 2022	Racial Disparities in Hepatitis B Birth Dose in the Washington Metropolitan Region, 2018-2020
Kattan 2014	Effect of vaccination coordinators on socioeconomic disparities in immunization among the 2006 Connecticut birth cohort.
Katz 2015	Does a pay-for-performance program for primary care physicians alleviate health inequity in childhood vaccination rates?
Kc 2017	Increased immunization coverage addresses the equity gap in Nepal.
Khan 2018	Exploring the spatial heterogeneity in different doses of vaccination coverage in India.
Khan 2020	Socioeconomic inequality trends in childhood vaccination coverage in India: Findings from multiple rounds of National Family Health Survey.
Khowaja 2015	Routine EPI coverage: subdistrict inequalities and reasons for immunization failure in a rural setting in Pakistan.
Kien 2017	Trends in childhood measles vaccination highlight socioeconomic inequalities in Vietnam.
Kirkby 2021	Subnational inequalities in diphtheria-tetanus-pertussis immunization in 24 countries in the African Region.
Kriss 2016	Vaccine receipt and vaccine card availability among children of the apostolic faith: analysis from the 2010-2011 Zimbabwe demographic and health survey.
Kulkarni 2021	Persistent Disparities in Immunization Rates for the Seven-Vaccine Series Among Infants 19-35 Months in the United States.
Kumar 2016	Socioeconomic disparities in coverage of full immunisation among children of adolescent mothers in India, 1990-2006: a repeated cross-sectional analysis.
Kunieda 2022	Individual- and Neighborhood-Level Factors of Measles Vaccination Coverage in Niamey, Niger: A Multilevel Analysis.
Lanaspa 2015	The performance of the expanded programme on immunization in a rural area of Mozambique.
Lawal 2023	Spatio-temporal analysis of childhood vaccine uptake in Nigeria: a hierarchical Bayesian Zero-inflated Poisson approach.
Lead author, year	Title
Lerm 2023	Inequalities in child immunization coverage: potential lessons from the Guinea-Bissau case.
Liu 2020	Vaccination coverage and its determinants of live attenuated hepatitis A vaccine among children aged 24-59 months in 20 rural counties of 10 provinces of China in 2016.
Lu 2021	Women's Empowerment and Children's Complete Vaccination in the Democratic Republic of the Congo: A Cross-Sectional Analysis.
Mak 2023	Multivariate assessment of vaccine equity in Nigeria: A VERSE tool case study using demographic and health survey 2018.
Marek 2020	Investigating spatial variation and change (2006-2017) in childhood immunisation coverage in New Zealand.
Marek 2021	Spatial-temporal patterns of childhood immunization in New Zealand (2006-2017): an improving pattern but not for all?

Masters 2019	Childhood vaccination in Kenya: socioeconomic determinants and disparities among the Somali ethnic community.
Mehmood 2022	Prevalence, geographical distribution and factors associated with pentavalent vaccine zero dose status among children in Sindh, Pakistan: analysis of data from the 2017 and 2018 birth cohorts enrolled in the provincial electronic immunisation registry.
Michels 2022	Evaluating vaccination coverage and timeliness in American Indian/Alaska Native and non-Hispanic White children using state immunization information system data, 2015-2017.
Michels 2023	Failure to Complete Multidose Vaccine Series in Early Childhood
Mishra 2020	Migration and child health: Understanding the coverage of child immunization among migrants across different socio-economic groups in India
Moran 2020	Socioeconomic characteristics associated with the introduction of new vaccines and full childhood vaccination in Ghana, 2014.
Mosser 2019	Mapping diphtheria-pertussis-tetanus vaccine coverage in Africa, 2000-2016: a spatial and temporal modelling study.
Muhoza 2023	Predictors for Uptake of Vaccines Offered during the Second Year of Life: Second Dose of Measles-Containing Vaccine and Meningococcal Serogroup A-Containing Vaccine, Ghana, 2020.
Mutua 2020	Do inequalities exist in the disadvantaged populations? Levels and trends of full and on-time vaccination coverage in two Nairobi urban informal settlements.
Mvula 2016	Predictors of Uptake and Timeliness of Newly Introduced Pneumococcal and Rotavirus Vaccines, and of Measles Vaccine in Rural Malawi: A Population Cohort Study.
Nagaoka 2016	Impact of Subsidies and Socioeconomic Status on Varicella Vaccination in Greater Tokyo, Japan.
Nchinjoh 2022	Factors Associated with Zero-Dose Childhood Vaccination Status in a Remote Fishing Community in Cameroon: A Cross-Sectional Analytical Study
Nda'chiDeffo 2020	Do the dynamics of vaccine programs improve the full immunization of children under the age of five in Cameroon?
Ndwandwe 2021	Incomplete vaccination and associated factors among children aged 12-23 months in South Africa: an analysis of the South African demographic and health survey 2016.
Nguyen 2023	Disparities in child and adolescent COVID-19 vaccination coverage and parental intent toward vaccinations for their children and adolescents.
NguyenSiAnh 2019	Hepatitis B Birth Dose Vaccination among Vietnamese Children: Implications for the Expanded Program on Immunization.
Nozaki 2019	Factors influencing basic vaccination coverage in Myanmar: secondary analysis of 2015 Myanmar demographic and health survey data
NtendaPAM 2017	Analysis of the effects of individual and community level factors on childhood immunization in Malawi.
Obanewa 2020	The role of place of residency in childhood immunisation coverage in Nigeria: analysis of data from three DHS rounds 2003-2013.
Ogundele 2022	Determinants of incomplete vaccination among children 12-23 months in Nigeria: An analysis of a national sample.
Oliveira 2014	Factors associated with vaccination coverage in children < 5 years in Angola.
Oster 2019	Sociodemographic, clinical and birth hospitalization characteristics and infant Hepatitis B vaccination in Washington State.
Oyefara 2014	Mothers' Characteristics and Immunization Status of Under-Five Children in Ojo Local Government Area, Lagos State, Nigeria
Pal 2016	Decomposing Inequality of Opportunity in Immunization by Circumstances: Evidence from India
Panda 2020	Understanding the full-immunization gap in districts of India: A geospatial approach
Patel 2021	Immunization status of children in Nepal and associated factors, 2016.

Patenaude 2022	A standardized approach for measuring multivariate equity in vaccination coverage, cost-of-illness, and health outcomes: Evidence from the Vaccine Economics Research for Sustainability & Equity (VERSE) project
Patenaude 2023	Comparing Multivariate with Wealth-Based Inequity in Vaccination Coverage in 56 Countries: Toward a Better Measure of Equity in Vaccination Coverage.
Peretti-Watel 2020	Determinants of childhood immunizations in Senegal: Adding previous shots to sociodemographic background.
Perry 2020	Timeliness and equity of infant pertussis vaccination in wales: Analysis of the three dose primary course.
Porth 2021	Women's Empowerment and Child Vaccination in Kenya: The Modifying Role of Wealth.
Portnoy 2020	Comparative Distributional Impact of Routine Immunization and Supplementary Immunization Activities in Delivery of Measles Vaccine in Low- and Middle-Income Countries.
Prusty 2014	Socioeconomic dynamics of gender disparity in childhood immunization in India, 1992-2006.
Rafferty 2019	Measurement of coverage, compliance and determinants of uptake in a publicly funded rotavirus vaccination programme: a retrospective cohort study.
Rahman 2018	Equity and determinants of routine child immunisation programme among tribal and non-tribal populations in rural Tangail subdistrict, Bangladesh: a cohort study.
Rajaonarifara 2022	Impact of health system strengthening on delivery strategies to improve child immunisation coverage and inequalities in rural Madagascar.
Rajpal 2023	Patterns in the Prevalence of Unvaccinated Children Across 36 States and Union Territories in India, 1993-2021.
Rammohan 2015	District-level variations in childhood immunizations in India: The role of socio-economic factors and health infrastructure.
Raza 2018	Differential achievements in childhood immunization across geographical regions of Pakistan: analysis of wealth-related inequality.
Restrepo-Méndez 2016	Inequalities in full immunization coverage: trends in low- and middle-income countries.
Roberts 2016	Measuring inequalities in immunization in Wales and the impact of interventions.
Roy 2023	A Decomposition Analysis to Understand the Wealth-Based Inequalities in Child Vaccination in Rural Southern Assam: A Cross-Sectional Study.
Saikia 2023	What Determines the District-Level Disparities in Immunization Coverage in India: Findings from Five Rounds of the National Family Health Survey.
Sandford 2015	Is there an association between the coverage of immunisation boosters by the age of 5 and deprivation? An ecological study.
Santorelli 2020	Factors associated with the uptake of the UK routine childhood immunization schedule in a bi-ethnic population
Santos 2022	Religious affiliation as a driver of immunization coverage: Analyses of zero-dose vaccine prevalence in 66 low- and middle-income countries.
Sarker 2019	Coverage and Determinants of Full Immunization: Vaccination Coverage among Senegalese Children.
Seck 2016	Social determinants of routine immunization coverage of children aged 72 to 23 months in the Kaolack region of Senegal
Setiawan 2022	Determinants of immunization status of children under two years old in Sumatera, Indonesia: A multilevel analysis of the 2020 Indonesia National Socio-Economic Survey.
Shaikh 2022	Gender differentials in spatial distribution of immunisation status in children aged 12-23 months by district in Punjab - Results from Pakistan Social and Living Standards Measurements Survey 2014-15.
Shanawaz 2014	An Evaluation Of Primary Immunization Coverage Among ICDS Children Under Urban Field Practice Area Of Osmania Medical College, Hyderabad
Sharma 2021	Income-based inequality in full immunization coverage of children aged 12-23 months in Eastern India: A decomposition analysis

Shenton 2018	Vaccination status of children aged 1-4â€¯years in Afghanistan and associated factors, 2015.
Shibre 2020	Inequalities in measles immunization coverage in Ethiopia: a cross-sectional analysis of demographic and health surveys 2000-2016.
Shiferie 2023	Vaccination dropout and wealth related inequality among children aged 12-35 months in remote and underserved settings of Ethiopia: a cross-sectional evaluation survey.
Shrivastwa 2015	Predictors of vaccination in India for children aged 12-36 months
Shrivastwa 2019	Analysis of State-Specific Differences in Childhood Vaccination Coverage in Rural India.
Siddiqi 2023	Immunization Gender Inequity in Pakistan: An Analysis of 6.2 Million Children Born from 2019 to 2022 and Enrolled in the Sindh Electronic Immunization Registry.
Siddiqui 2014	Ethnic disparities in routine immunization coverage: a reason for persistent poliovirus circulation in Karachi, Pakistan?
Singh 2014	Sibling composition and child immunization in India and Pakistan, 1990-2007.
Singh 2015	Gender based within-household inequality in immunization status of children: some evidence from South Asian countries
Singh 2019	Immunization coverage among children aged 12-23 months: A cross sectional study in low performing blocks of Bihar, India.
Singh 2020	Gender discrimination and other factors affecting Full Immunization Coverage (FIC) in 59 low performing blocks of Bihar
Siramaneerat 2021	Inequalities in immunization coverage in Indonesia: a multilevel analysis.
Sissoko 2014	The Influence of Compositional and Contextual Factors on Non-Receipt of Basic Vaccines among Children of 12-23-Month Old in India: A Multilevel Analysis
Smalley 2023	Factors associated with vaccine coverage improvements in Senegal between 2005 and 2019: a quantitative retrospective analysis.
Song 2020	Inequalities in complete childhood immunisation in Nepal: results from a population-based cross-sectional study.
Soura 2015	Understanding inequities in child vaccination rates among the urban poor: evidence from Nairobi and Ouagadougou health and demographic surveillance systems.
Sowe 2019	Disentangling the rural-urban immunization coverage disparity in The Gambia: A Fairlie decomposition.
Sowe 2022	Does a pay-for-performance health service model improve overall and rural-urban inequity in vaccination rates? A difference-in-differences analysis from the Gambia.
Srivastava 2020	Explaining socioeconomic inequalities in immunisation coverage in India: new insights from the fourth National Family Health Survey (2015-16).
Srivastava 2022	Socioeconomic inequalities in non- coverage of full vaccination among children in Bangladesh: a comparative study of Demographic and Health Surveys, 2007 and 2017-18.
Taneja 2023	An Equity Aanlysis of Zero-Dose Children in India Using the National Family Health Survey Data Status, Challenges and Next Steps
Tesfa 2023	Spatial distribution of complete basic childhood vaccination and associated factors among children aged 12-23 months in Ethiopia. A spatial and multilevel analysis.
Tessier 2018	Population-level factors predicting variation in influenza vaccine uptake among adults and young children in England, 2015/16 and 2016/17.
Thapa 2021	Associated Factors for Dropout of First Vs Third Doses of Diphtheria Tetanus Pertussis (DPT) Vaccination in Nepal.
Tiley 2022	Equity of the Meningitis B vaccination programme in England, 2016-2018.
Tola 2020	High Inequality and Slow Services Improvement in Newborn and Child Health Interventions in Ethiopia.
Tur-Sinai 2019	Vaccination uptake and income inequalities within a mass vaccination campaign.

Utazi 2022	Assessing the characteristics of un- and under-vaccinated children in low- and middle-income countries: A multi-level cross-sectional study.
Uthman 2017	Children who have received no routine polio vaccines in Nigeria: Who are they and where do they live?
Uzochukwu 2017	Inequity in access to childhood immunization in Enugu urban, Southeast Nigeria.
Varan 2017	Vaccination Coverage Disparities Between Foreign-Born and U.S.-Born Children Aged 19-35Â Months, United States, 2010-2012.
Vo 2019	Trends in Socioeconomic Inequalities in Full Vaccination Coverage among Vietnamese Children aged 12-23 Months, 2000-2014: Evidence for Mitigating Disparities in Vaccination.
Vyas 2019	Understanding Spatial and Contextual Factors Influencing Intraregional Differences in Child Vaccination Coverage in Bangladesh.
Wagner 2014	Childhood vaccination coverage by ethnicity within London between 2006/2007 and 2010/2011.
Wahl 2021	Change in full immunization inequalities in Indian children 12-23â€‰months: an analysis of household survey data.
Walker 2014	Reduction of racial/ethnic disparities in vaccination coverage, 1995-2011.
Walsh 2016	Since The Start Of The Vaccines For Children Program, Uptake Has Increased, And Most Disparities Have Decreased.
Wandera 2017	Variation in rotavirus vaccine coverage by sub-counties in Kenya.
Wariri 2019	Tracking coverage, dropout and multidimensional equity gaps in immunisation systems in West Africa, 2000-2017.
Wendt 2021	Are children in female-headed households at a disadvantage? An analysis of immunization coverage and stunting prevalence: in 95 low- and middle-income countries.
Wendt 2022	Children of more empowered women are less likely to be left without vaccination in low- and middle-income countries: A global analysis of 50 DHS surveys.
Wendt 2022	Exposure of Zero-Dose Children to Multiple Deprivation: Analyses of Data from 80 Low- and Middle-Income Countries.
Win 2022	Equity assessment of childhood immunisation at national and subnational levels in Myanmar: a benefit incidence analysis.
Woinarowicz 2020	Comparing vaccination coverage of American Indian children with White children in North Dakota.
Wolf 2016	Parental Country of Birth and Childhood Vaccination Uptake in Washington State.
Wondimu 2020	Inequalities in Rotavirus Vaccine Uptake in Ethiopia: A Decomposition Analysis.
Wondimu 2021	Persistent Socioeconomic Inequalities in Measles Vaccine Uptake in Ethiopia in the Period 2005 to 2016.
Xeuatvongsa 2017	Determination of factors affecting the vaccination status of children aged 12-35 months in Lao People's Democratic Republic.
Yakum 2023	Factors associated with full vaccination and zero vaccine dose in children aged 12-59Â months in 6 health districts of Cameroon.
Yibeltal 2022	Trends, projection and inequalities in full immunization coverage in Ethiopia: in the period 2000-2019.
Yingtaweesak 2021	Socioeconomic Factors Affecting Vaccination Coverage And Timeliness Among Children Living Along The Thai-Myanmar Border
Zegeye 2021	Demographic and health surveys showed widening trends in polio immunisation inequalities in Guinea.
Zhang 2018	Rural-urban disparity in category II vaccination among children under five years of age: evidence from a survey in Shandong, China.
Zhang 2019	Uptake of maternal care and childhood immunization among ethnic minority and Han populations in Sichuan province: a study based on the 2003, 2008 and 2013 health service surveys.

Zhang 2022	Coverage and Equity of Childhood Vaccines in China.
Zhang 2023	Ethnic inequities in routine childhood vaccinations in England 2006-2021: an observational cohort study using electronic health records.
Zhao 2023	Multivariate Assessment of Vaccine Equity in Cambodia: A Longitudinal VERSE Tool Case Study Using Demographic and Health Survey 2004, 2010, and 2014.
