# Supplementary material for: Inequality in Childhood Immunization Coverage: A Scoping Review of Data Sources, Analyses, and Reporting Methods
Source: Vaccines (Basel). 2024 Jul 29;12(8):850. doi: 10.3390/vaccines12080850 (PMC11360733; doi:10.3390/vaccines12080850)
Supplement: Supplementary file 1 [file vaccines-12-00850-s001.zip › vaccines-3083542 - Supplementary Tables S2, S3.pdf]

Table S2. Summary of findings of single-country studies on inequalities in child vaccine coverage conducted between 2013-2023

PROGRESS-Plus characteristic	Full coverage of multiple vaccines (N=121)
Place of residence (rural/urban) (N= 73)	30 studies reported greater urban coverage [46-74]<br>15 studies reported greater rural coverage [45, 75-87]<br>7 studies found inconsistent or mixed associations [37, 88-94]<br>21 studies found no, minimal or statistically insignificant difference by place of residence [71, 95-114]
Race, ethnicity, culture, language<br>(N= 56)	49 studies* show pattern of significant relationship between race, ethnicity, culture and language, in most cases lower coverage of ethnic and racial and linguistic minorities (of these one did not test statistical significance, and one other found that ethnic minorities had greater coverage)<br>7 studies found no association or associations that were not statistically significant [81, 82, 95, 106, 115-117]
Maternal Occupation<br>(N= 33)	10 studies found greater coverage for children with employed mothers[45, 48, 67, 71, 78, 106, 109, 118-120]<br>6 studies found greater coverage for children with unemployed mothers – of these 3 were in China[71, 75, 84, 114, 121, 122]<br>7 studies reported variation by type of maternal occupation or having employment[50, 63, 83, 86, 117, 123]<br>10 studies found no association[52, 68, 69, 93, 95, 97, 101, 104, 124, 125]
Paternal Occupation<br>(N= 9)	Three found no significant association,[97, 101, 104] while six found a significant association[63, 69, 83, 116, 118, 123]
Gender and sex<br>(N= 66)	11 studies found higher coverage among male children[55, 58, 59, 115, 116, 126-129]<br>2 studies found higher coverage among female children[57, 125]<br>49 studies found no statistically significant association<br>4 studies were inconclusive
Religion<br>(N= 38)	10 studies found lower coverage among non-majority Muslim faith and religious groups[37, 58-60, 116, 126, 128-130]; one study reported greater odds of immunization among non-migrant religious groups[74]<br>3 studies found higher coverage among Christian faith groups as compared to other (minority or majority groups) [48, 91, 131] while one study found that a particular sect of Christianity had lower coverage compared to others[113]<br>13 studies found significant variation by religion[56, 61, 62, 81, 86, 98, 99, 120, 125, 127, 128, 132, 133]<br>2 studies found that having no religion was associated with lower coverage[78, 118]<br>10 studies found no statistically significant or minimal association[34, 52, 66, 79, 80, 89, 102, 115, 117, 134]
Education (maternal)<br>(N= 100)	79 studies* showed greater coverage among those who were literate or those with higher education<br>17 studies showed no statistically significant association[45, 52, 56, 68, 69, 72, 79, 86, 95, 104, 105, 111, 116, 120, 125, 134-137]<br>4 studies showed the contribution of education to other inequalities related to wealth[56, 89, 138] and caste/ethnic disparities[92]
Socioeconomic status<br>(N= 94)	75 studies* showed greater coverage among higher income or wealth groups, or lower coverage among poorer groups<br>13 studies showed no association or non-statistical significance of economic status[54, 56, 79, 95, 97, 106, 110, 134-137]<br>6 studies showed mixed or changing associations, or non-significant results at individual versus ecological levels[44, 93, 103, 109, 114, 139]

Subnational region (N=49)	47 studies showed significant variation by subnational region<br>2 studies reported no significant association[106, 136]

Table S3. Summary of findings of multi-country studies on inequalities in child vaccine coverage conducted between 2013-2023

**PROGRESS-Plus characteristic**	**Any vaccine outcome (N=39)**
Place of residence (rural/urban) (N=4)	Three of these showed greater coverage in urban areas[33, 140, 141], and one found lower coverage in urban areas[142]
Race, ethnicity, culture, language (N=3)	Three studies found some association of ethnicity with coverage, with varying patterns across countries[123, 142, 143].
Occupation (N=3)	Employment, particularly non-agricultural employment, was associated with greater coverage[123, 141, 144]
Gender and sex (N=10)	Child sex was found not to have an association with full coverage in a number of studies[90, 123, 142, 144-146] although two studies did find lower coverage among females[140, 147], while others found variation[148, 149]
Religion (N=2)	Santos et al[150] found that minority religions (Muslim, folk, unaffiliated) were more likely to be unvaccinated as compared to majority populations, while Utazi et al[90] found inconsistent associations across the 9 countries assessed.
Education (maternal) (N=16)	Evidence suggesting a role of education in increasing vaccine coverage was substantial and significant[33, 40, 90, 123, 140-142, 144, 145, 148, 151-156].
Socioeconomic status (N=27)	With the exception of one study[157], economic status was consistently associated with gains and greater levels of full vaccine coverage across 25 studies that reported this dimension of inequality.<br>Wendt et al[156] utilized a deprivation index and found that the greater the deprivation, the lower the vaccine coverage.
